# Age Related Changes in Topological Properties of Brain Functional Network and Structural Connectivity

**DOI:** 10.3389/fnins.2018.00318

**Published:** 2018-05-15

**Authors:** Chandan Shah, Jia Liu, Peilin Lv, Huaiqiang Sun, Yuan Xiao, Jieke Liu, Youjin Zhao, Wenjing Zhang, Li Yao, Qiyong Gong, Su Lui

**Affiliations:** ^1^Huaxi MR Research Center, Department of Radiology, West China Hospital of Sichuan University, Chengdu, China; ^2^Department of Anesthesiology, West China Hospital of Sichuan University, Chengdu, China

**Keywords:** healthy participants, magnetic resonance imaging, topological properties, fractional anisotropy, white matter

## Abstract

**Introduction:** There are still uncertainties about the true nature of age related changes in topological properties of the brain functional network and its structural connectivity during various developmental stages. In this cross- sectional study, we investigated the effects of age and its relationship with regional nodal properties of the functional brain network and white matter integrity.

**Method:** DTI and fMRI data were acquired from 458 healthy Chinese participants ranging from age 8 to 81 years. Tractography was conducted on the DTI data using FSL. Graph Theory analyses were conducted on the functional data yielding topological properties of the functional network using SPM and GRETNA toolbox. Two multiple regressions were performed to investigate the effects of age on nodal topological properties of the functional brain network and white matter integrity.

**Result:** For the functional studies, we observed that regional nodal characteristics such as node betweenness were decreased while node degree and node efficiency was increased in relation to increasing age. Perversely, we observed that the relationship between nodal topological properties and fasciculus structures were primarily positive for nodal betweenness but negative for nodal degree and nodal efficiency. Decrease in functional nodal betweenness was primarily located in superior frontal lobe, right occipital lobe and the global hubs. These brain regions also had both direct and indirect anatomical relationships with the 14 fiber bundles. A linear age related decreases in the Fractional anisotropy (FA) value was found in the callosum forceps minor.

**Conclusion:** These results suggests that age related differences were more pronounced in the functional than in structural measure indicating these measures do not have direct one-to-one mapping. Our study also indicates that the fiber bundles with longer fibers exhibited a more pronounced effect on the properties of functional network.

## Introduction

Aging is considered as one of the greatest risk factors for neurodegenerative diseases where there is progressive loss of either structure or function of the neurons (Morrison and Hof, 1997). It is well-stipulated that marked transformations in white matter structure occur as early as third trimester (Ball et al., 2014) which persists throughout childhood and adolescence (Lebel et al., 2008; Hagmann et al., 2010; Tamnes et al., 2010; Dubois et al., 2014; van den Heuvel et al., 2015; Wierenga et al., 2016). These transformations reflects an increase in axonal conduction speed (Baumann and Pham-Dinh, 2001), thereby resulting in improvement of information transfer (van der Knaap et al., 1991). Similarly, functional studies have also investigated the effects of age on functional brain connectivity showing older adults to have lower functional connectivity between regions of the default mode network compared to younger adults (Ferreira and Busatto, 2013; Dennis and Thompson, 2014). However, little is known about these transformations beyond the young adults until the sixth or seventh decades of life, which is more vulnerable to the effects neurodegenerative diseases that show its effects abruptly after a possible long preclinical period (Grady et al., 1988; Berg et al., 1992; Morris et al., 1993). Although recent advances in network analysis have provided new insights into both structural and functional connectivity pattern, which is considered as the core of the brain activity (Sporns et al., 2005; Bullmore and Bassett, 2011; Sporns, 2012), it is still not fully understood about the relationships between them across lifespan, the evaluation of which might help us elucidate the possible mechanisms behind age related neurodegenerative diseases.

Among the broad range of network analysis approaches, graph theory approach is considered one of the favorable as it applies to both structural and functional connectivity, which is a natural framework for the exact mathematical representation of complex networks. In graph theory, a network is defined as a set of nodes with edges between them. Some frequently used network measures include: global network properties such as small-world properties [clustering coefficient (Cp) and characteristics path length (Lp)]; efficiency metrics (global efficiency and local efficiency); and regional nodal properties (nodal betweenness, nodal degree, and nodal efficiency) (Bullmore and Sporns, 2009). Similarly, patterns of *in vivo* structural brain connectivity can be investigated by using diffusion tensor imaging (DTI), which is a MRI modality based on principle of water diffusion measures (Beaulieu, 2002). One of the most frequently examined aspects of structural connectivity is fractional anisotropy (FA), where higher FA is interpreted as greater structural connectivity between regions.

In recent past, increasing number of studies have used graph theory to describe large scale topological organization of various structural and functional brain networks such as small world property, network efficiency, modular structure, and rich club architecture (Achard et al., 2006; He et al., 2007; Bassett and Bullmore, 2009; Stam, 2010; van den Heuvel and Sporns, 2011, 2013). Using a graph theoretical approach Wierenga and colleagues measured whole brain connectivity in childhood and adolescence and observed a sequential maturational model where they observed that connections between unimodal regions strengthen in childhood, followed by connection of these unimodal regions to association regions, adolescence was characterized by the strengthening of connections between association regions within the frontal and parietal cortex (Wierenga et al., 2016). In fact, their studies showed that white matter strengthening was not homogenous throughout the brain during childhood and adolescence showing strengthening of short association fibers rather than long association fibers. Interestingly, Cao and colleagues in their study (Cao et al., 2014) found an increase in network efficiency during early adulthood which showed a decreasing trend with increasing age. They also observed the proportions of short-distance fibers to be higher than those of long-distance fibers in older adults did. This was also supported by the findings of Sala-Lincon and colleagues, who observed higher average clustering coefficient as well as higher shortest path length with increasing age (Sala-Llonch et al., 2014). Similarly, age related differences in structural connectivity have also observed wide–spread decreases in fractional anisotropy in older compared to younger adults (Damoiseaux and Greicius, 2009; Burzynska et al., 2010) which is also supported by other studies (Gong et al., 2009b; Otte et al., 2015; Zhao et al., 2015). It can therefore be asserted that, although strong functional connectivity between brain regions can exist in absence of strong structural connectivity (Damoiseaux and Greicius, 2009; Zimmermann et al., 2016), similar patterns of age related differences in structural and functional connectivity metrics greatly suggests probable association between these two measures (Andrews-Hanna et al., 2007; Betzel et al., 2014; Fjell et al., 2016; Zimmermann et al., 2016).

Despite of many advances in neuroscience, there is still much to learn about the relationship between white matter integrity and nodal topological properties of structural and functional network. So far, Cao et al. (2014) has employed graph theory across life span ranging 7–85 years. However, they have only used resting state functional fMRI data to examine the topological age related effects. Other study by Hirsiger et al. (2016) (*n* = 165 age range 64–85) have examined the association of structural and functional connectivity of the cingulum bundle to probe the cognitive and motor performance, where they have suggested that only structural connectivity but not resting state functional connectivity was significantly associated with age. Since, the characterization on structural and functional connectivity is essential to determine the relationship between them. We, therefore have made an effort to ascertain the nature of functional connections by examining the strength of structural connections and vice-versa in a large sample of healthy subjects (*n* = 458) with an age range of 8–81 years. We hypothesize that changes in both white matter integrity and functional connectivity would be observed in certain networks, and these changes in nodal topological properties of the functional network would be associated with the changes in FA.

## Materials and methods

### Participants

DTI and fMRI data were acquired on 3T MRI systems from 458 healthy Chinese Han subjects (right handed; 229 males, 229 females; age range, 8–81 years) at West China Hospital of Sichuan University. The subjects were recruited via local advertising. Among these, 443 participants had fMRI data, 346 participants had DTI data, and 331 participants had both fMRI and DTI data (Table 1). Participants had no history of brain injury, neurological, or psychiatric diseases. Informed written consent was obtained from all participants including parent/guardian consent for younger participants. Research protocol was approved by the local ethics committee.

**Table 1:** Participant characteristics.

**Characteristics**	**Total**		**fMRI**		**DTI**		**fMRI** + **DTI**	
	**Male**	**Female**	**Male**	**Female**	**Male**	**Female**	**Male**	**Female**
Number of individuals, n	229	229	219	224	174	172	164	167
Age, y (mean)	27.57	29.54	27.47	29.71	28.75	30.70	28.70	30.97
Education, y (mean)	13.70	12.77	13.73	12.74	13.72	12.71	13.76	12.66

### Data acquisition

All subjects were scanned in a single session without changing their position and were instructed to remain motionless as possible and relax their minds with their eyes open. All participants confirmed they remained awake and alert throughout the scanning session.

### Functional MRI

The subjects underwent a resting-state fMRI scan in one of two 3T MRI systems [General Electric (EXCITE, Millwaukee, USA) or Siemens (Trio a Tim, Erlangen, Germany)]. Parameters of General Electric machine were; repetition time = 2,000 ms, echo time = 30 ms, field of view (FOV) = 24 × 24 cm^2^, flip angle = 90°, slice thickness = 5.0 mm (no gap), voxel size, 3.75 × 3.75 × 5 mm^3^; matrix, 64 × 64. Parameters of Siemens machine were: repetition time = 2,000 ms, echo time = 30 ms, field of view (FOV) = 24 × 24 cm^2^, flip angle = 90°, slice thickness = 5.0 mm (no gap), voxel size 3.75 × 3.75 × 5 mm^3^, matrix 64 × 64.

### Diffusion tensor imaging

DTI data were also acquired from one of two 3T MRI systems. Parameters of General Electric machine were: TR = 10,000 ms, TE = 70.8 ms, field of view (FOV) = 24 × 24 cm^2^, resolution matrix 128 × 128, flip angle = 90°, slice thickness = 3.0 mm, A total of 672 slices were acquired for b values of b = 0 and b = 1,000 mm^2^/s, which were obtained by applying gradients along 15 non-collinear directions. Parameters of Siemens machine were; TR = 6,800 ms, TE = 93 ms, field of view (FOV) = 24 × 24 cm^2^, resolution matrix 128 × 128, flip angle = 90°, slice thickness = 3.0 mm. 42 slices were acquired for b = 0 and b = 1,000 mm^2^/s; these were obtained by applying gradients along 30 non-collinear directions.

### Data preprocessing

Preprocessing contained both functional and structural preprocessing steps.

Functional preprocessing was carried out using Statistical Parametric Mapping (SPM 8) (SPM8, http://www.fil.ion.ucl.ac.uk/spm) and GRETNA (He et al., 2008). Briefly, preprocessing was done by (i) discarding first 10 functional volumes for signal equilibration, (ii) slice timing correction for timing offsets, (iii) head motion correction by 3D geometrical displacement, and (iv) normalization to Montreal Neurological Institute (MNI) space. All data used in this study satisfied the criteria of spatial movement in any direction <1.5 mm or degree. Subjects demonstrated no significant group differences in head-motion parameters. Furthermore, linear detrend and band-pass filtering (0.01–0.08 Hz) was performed to reduce the effects of low-frequency drift and high-frequency noise. Subsequently, several nuisance signals including head motion, global mean, and signals from the cerebrospinal fluid and white matter were regressed from the data. For structural preprocessing, raw DTI images were preprocessed using the FSL (FMRIB Software Library, FMRIB, Oxford, UK) (Smith et al., 2004) software package. For each DTI dataset, all diffusion weighted images were affinely coregistered to the b0 image using FLIRT (FMRIB's Linear Image Registration Tool) (Jenkinson and Smith, 2001) with 12 degrees of freedom to correct for eddy current-induced distortion and subtle head motion. Brain mask was created from the b0 image using the BET (Brain extraction Tool) (Smith, 2002) with a fractional intensity threshold of 0.2; FDT (FMRIB's Diffusion Toolbox) (Behrens et al., 2003) was used to fit the tensor model.

**Figure 1:**
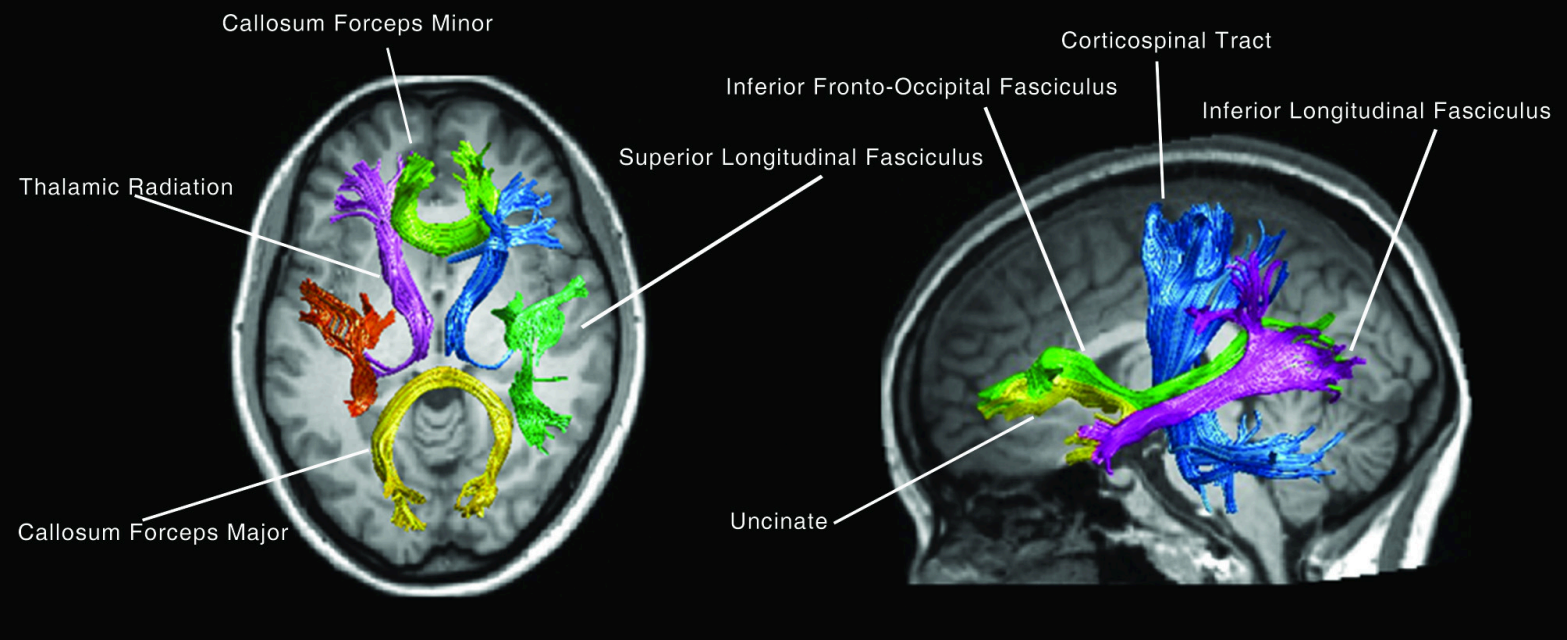
The 14 main fiber bundles.

For fiber tract identification, a MATLAB-based open source software termed “automatic fiber quantification (AFQ),” which implements both algorithms proposed by Hua and Zhang (Hua et al., 2008; Zhang et al., 2008) was used. Identification procedure included three primary steps; firstly, whole-brain fiber trajectory was performed on the preprocessed tensor images. Secondly, fiber tract segmentation was performed based on the waypoint region of interest (ROI) procedure as described by Wakana et al. (2007). The waypoint ROIs set developed in Mori's lab was warped into individual space from the MNI template space via non-linear transformation. Each fiber was defined as a candidate to a particular fiber group if it crossed through two-way point ROIs that were used to define a specific fiber tract. Thirdly, fiber refinement was accomplished by comparing each candidate fiber to the fiber tract probability map proposed by Hua et al. (2008). Fiber tract probability maps were also transformed into an individual's native space; candidate fibers for a particular fiber groups were assigned scores according to the probability values of the voxels that they passed through. Candidate fibers with low scores were discarded. Additionally, an iterative procedure was used to filter fibers that were aberrantly longer than the mean fiber length or that were distant from the core of the fiber tract. Fourteen fiber bundles were identified according to the predefined ROIs and probability maps. These bundles were the bilateral thalamic radiation, corticospinal tract, inferior fronto-occipital fasciculus, inferior longitudinal fasciculus, superior longitudinal fasciculus as well as the uncinate, arcuate, genu and splenium of the corpus callosum (Figure 1).

### Construction of functional brain network

Topological properties of corresponding brain networks were examined using the GRETNA toolbox. Small world behavior was assessed by coefficient σ (Humphries et al., 2006) which uses a ratio of network clustering and path length to contrast with the same metrics from an equivalent random network. For σ > 1, a network is considered to have small world behavior (Yang et al., 2015). Brain areas (except pons and cerebellum) were parceled into 90 brain regions according to the Automated Anatomical Labeling atlas (Tzourio-Mazoyer et al., 2002) and were used to define the nodes of the functional network. Then, global network properties including small-world properties, efficiency metrics, and regional nodal properties were simultaneously calculated. Briefly, small-world network is an anatomical network containing specialized (segregated) modules and a robust number of intermodular (integrating) links, that has clustering coefficient (Cp), and characteristics path length (Lp) as its properties. Some of the other metrics frequently used are efficiency metrics such as global efficiency (which represents the inverse of the average shortest path length between nodes in the entire network) and local efficiency (which represents the inverse of the average shortest path length between all nearest neighbors of a node). Regional nodal properties such as nodal betweenness (an indicator of a nodes centrality in a network, which is equal to the number of shortest paths, form all vertices to all others that pass through a node). Nodal degree (the degree of an individual node, which is equal to the number of links connected to that node), and nodal efficiency (the average smallest path weight between a given node and all other nodes in the network) (Bullmore and Sporns, 2009). Equations used to calculate these metrics could be found elsewhere (Rubinov and Sporns, 2010).

### Statistical analysis

All statistical analyses were performed using SPSS (version 19). Two multiple regressions were performed to investigate the effects of age on nodal topological properties of the functional brain network and white matter integrity with nodal topological properties and mean FA values as dependent variables while; age, gender, and education kept as independent variables. Additionally, multiple regression model was also used to explore the association between nodal topological properties and white matter integrity by using the value of each nodal topological property as an independent variable and mean FA value of each fiber bundle, maintaining age, gender, and education as dependent variables. Finally, two subgroup analyses were performed to investigate the interactive effect between gender and nodal topological properties or white matter integrity.

#### Linear regression of topological properties in the functional brain network according to age

We performed linear regression to analyze the effect of age on the topological properties (global network properties and regional network properties as dependent variables with statistical threshold set as *P* < 0.05).

#### Linear regression of FA value and age

At first, 14 fiber bundles from the DTI data and 100 nodal FA values of each fasciculus (Figure 1), then the mean FA values for each of the 14 fiber bundles were calculated. Finally, linear regression was performed to explore the effect of age on white matter integrity with mean FA of each fiber bundle as the dependent variable.

#### Linear regression of nodal topological properties according to mean FA value

Linear regression model was also used to explore the association between nodal topological properties and white matter integrity. While, the mean FA value of each fiber bundle was used as the independent variable, the value of each nodal topological property was used as the dependent variable.

## Results

### Linear regression of the topological properties of the functional brain network according to age

**Figure 2:**
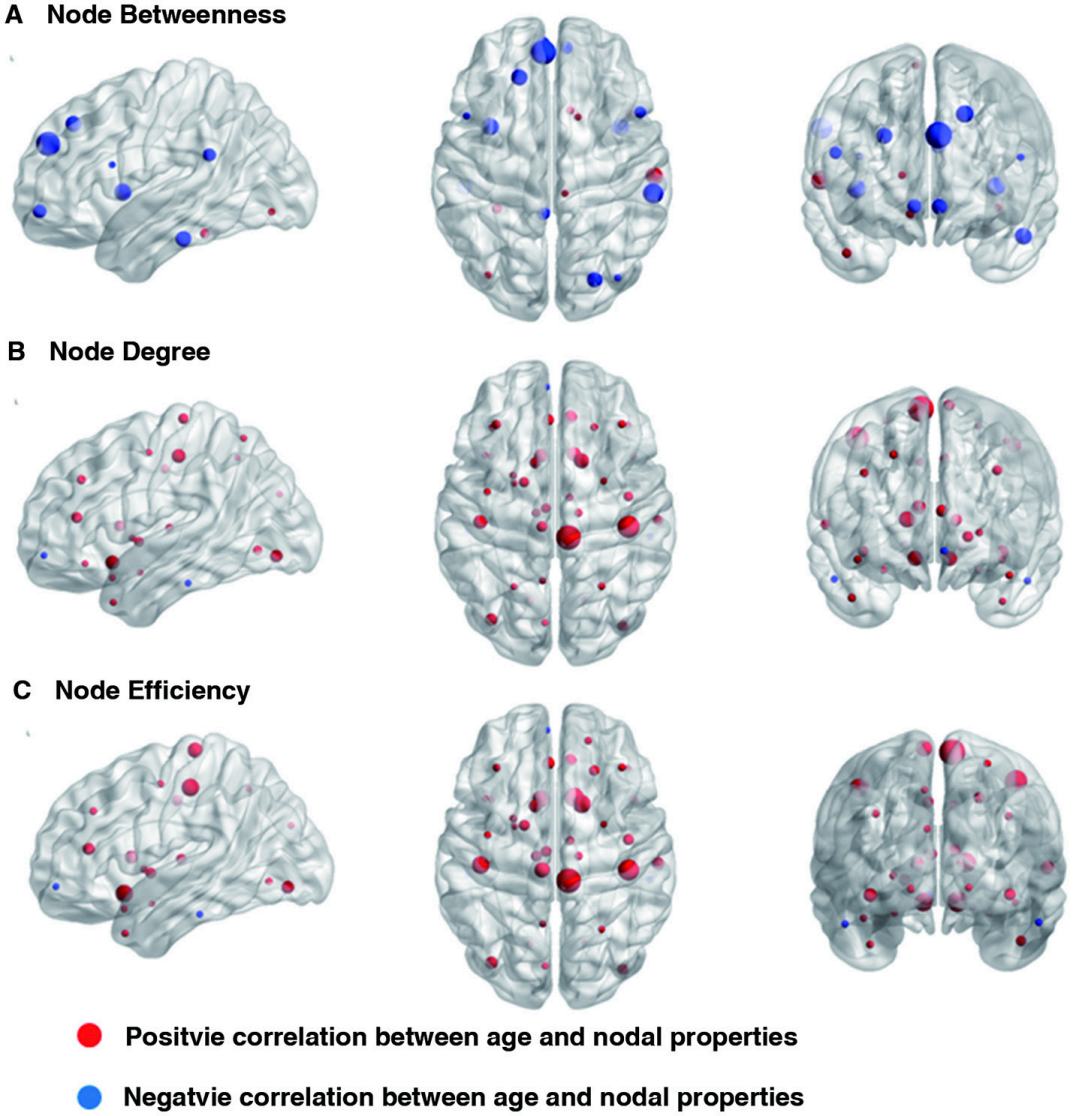
Effect of age on regional nodal properties. Significant positive and negative correlations between age and regional nodal parameters are shown in red and blue, respectively. The node sizes indicate the values of the regional nodal parameters. The distributions of nodes showing altered regional properties were visualized with the Brain Net Viewer (http://www.nitrc.org/projects/bnv/). **(A)** Node betweenness, **(B)** Node degree, and **(C)** Node efficiency.

**Figure 3:**
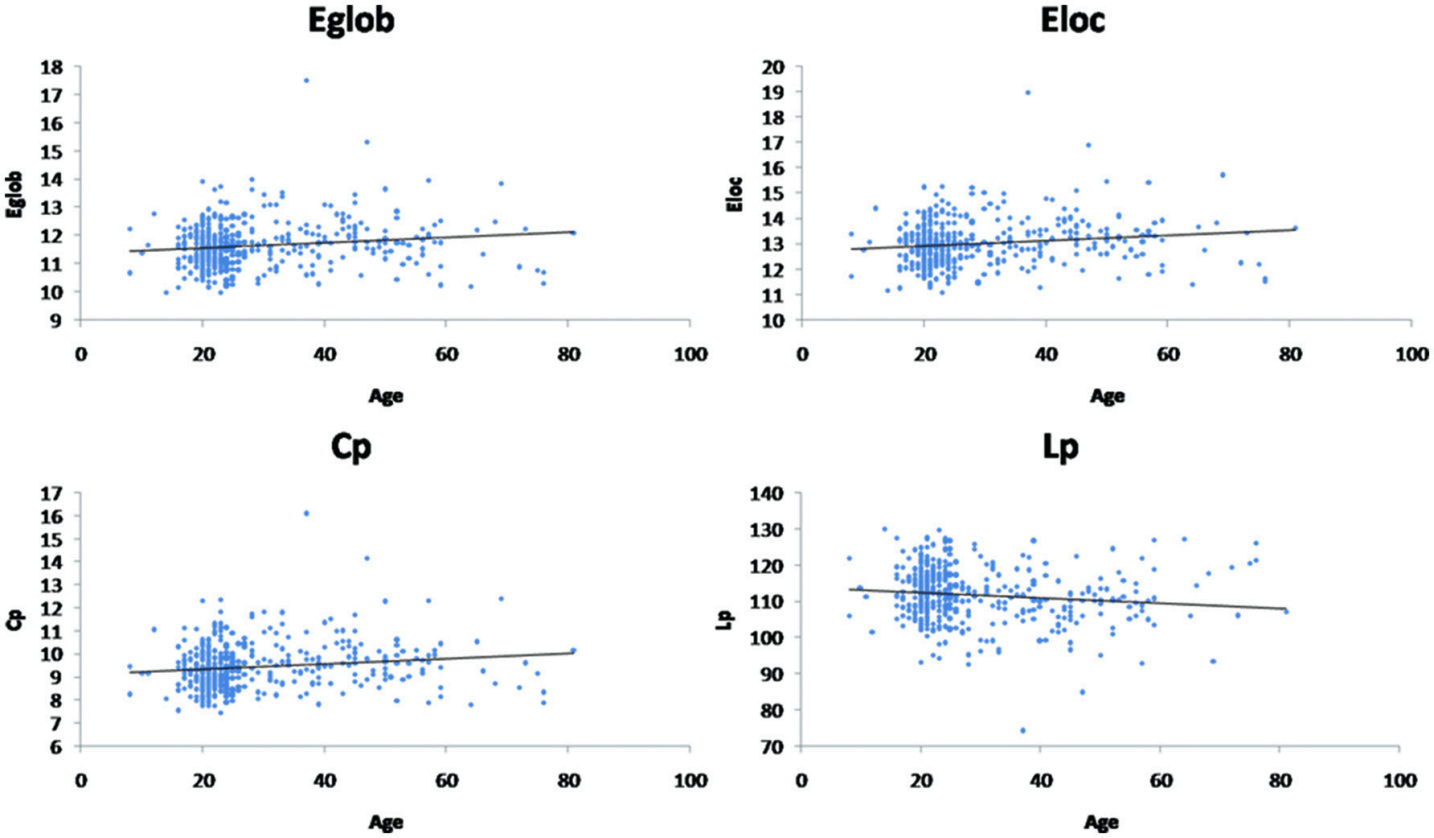
Effect of age on global network properties. Age is positively correlated with normalized global efficiency (Eglob) (β = 0.009, *p* = 0.004), local efficiency (Eloc) (β = 0.01, *p* = 0.002), and clustering coefficient (Cp) (β = 0.011, *p* = 0.002). Age is negatively correlated with path length (Lp) (β = −0.074, *p* = 0.004).

Linear age-related changes in the node betweenness were revealed in 21 brain regions. Positive correlation between age and node degree and node efficiency was observed in bilateral middle and inferior frontal gyrus, bilateral anterior and median cingulated cortex, bilateral postcentral gyrus, superior parietal gyrus, bilateral superior temporal gyrus, middle temporal gyrus, bilateral caudate nucleus and left lenticular nucleus (Table 2, Figures 2B,C, red regions). In addition, negative correlation between age and node degree and node efficiency was observed in bilateral inferior temporal gyrus and medial superior frontal gyrus (Figures 2B,C, blue regions). Likewise, age showed positive correlation with node betweenness in olfactory cortex, fusiform gyrus, superior and middle temporal gyrus (Figure 2A, red regions). However, negative correlation was observed in bilateral inferior and frontal gyrus; left superior frontal gyrus, bilateral insula, right superior and middle occipital gyrus (Figure 2A, blue regions). Figure 3 shows the linear regression results of the effects of age on the small-world properties and efficiency metrics wherein, linear positive age-related changes (*p* < 0.05) were calculated for global efficiency (β = 0.009, *p* = 0.004), local efficiency (β = 0.01, *p* = 0.002), and the clustering coefficient (β = 0.011, *p* = 0.002). A linear age-related reduction in path length was also observed (β = −0.074, *p* = 0.004).

**Table 2:** Regression models assessing the effect of age on nodal topological properties.

**Brain Regions**	**Betweenness**		**Degree**		**Node efficiency**	
	**Beta**	***p*-Value**	**Beta**	***p*-Value**	**Beta**	***p*-Value**
Precentral_L			1.462	0.044	0.012	0.038
Precentral_R			2.112	0.006	0.019	0.003
Frontal_Sup_L	−11.329	0.003				
Frontal_Sup_R			1.889	0.01	0.017	0.004
Frontal_Sup_Orb_R					0.015	0.014
Frontal_Mid_L			2.071	0.003	0.015	0.007
Frontal_Mid_R			1.901	0.01	0.015	0.016
Frontal_Inf_Oper_L	−6.128	0.045				
Frontal_Inf_Oper_R	−9.171	0.017				
Frontal_Inf_Orb_L			1.379	0.042		
Frontal_Inf_Orb_R			1.84	0.013	0.014	0.024
Olfactory_L			2.932	<0.001	0.026	<0.001
Olfactory_R	7.877	0.002	2.977	<0.001	0.028	<0.001
Frontal_Sup_Medial_L	−14.071	<0.001				
Frontal_Mid_Orb_L	−9.987	0.023	−1.588	0.034	−0.014	0.022
Frontal_Mid_Orb_R	−9.746	0.032				
Insula_L	−13.31	0.012				
Insula_R	−11.847	0.013				
Cingulum_Ant_L			2.654	<0.001	0.02	<0.001
Cingulum_Ant_R			2.738	<0.001	0.02	<0.001
Cingulum_Mid_L			2.175	0.004	0.018	0.003
Cingulum_Mid_R			1.896	0.012	0.016	0.011
Cingulum_Post_L	−9.264	0.01				
Amygdala_L			1.522	0.026	0.011	0.043
Amygdala_R			1.497	0.026	0.011	0.048
Cuneus_L			1.525	0.046	0.012	0.046
Lingual_L			2.133	0.01	0.015	0.033
Lingual_R	7.06	0.024	1.789	0.029	0.011	0.045
Occipital_Sup_R	−10.133	0.005				
Occipital_Mid_R	−6.322	0.029				
Occipital_Inf_L	6.374	0.012	2.711	0.001	0.021	0.003
Occipital_Inf_R			2.433	0.004	0.019	0.009
Fusiform_L	9.026	0.016				
Postcentral_L			2.813	0.001	0.025	<0.001
Postcentral_R			3.376	<0.001	0.029	<0.001
Parietal_Sup_L			1.421	0.005		
Parietal_Sup_R			1.834	0.008	0.013	0.024
SupraMarginal_R	−13.633	0.002				
Precuneus_L			1.632	0.014	0.012	0.024
Paracentral_Lobule_L			2.499	0.002	0.023	0.001
Paracentral_Lobule_R	4.849	0.014	3.673	<0.001	0.032	<0.001
Caudate_L			2.569	<0.001	0.021	<0.001
Caudate_R	5.111	0.047	3.109	<0.001	0.027	<0.001
Putamen_L			1.738	0.014	0.015	0.01
Pallidum_L			2.243	0.002	0.019	0.002
Thalamus_L			1.991	0.013	0.019	0.006
Thalamus_R			1.942	0.017	0.016	0.022
Temporal_Sup_R	13.414	<0.001	2.384	<0.001	0.021	<0.001
Temporal_Pole_Sup_L			1.637	0.022	0.013	0.03
Temporal_Pole_Mid_L			1.495	0.037	0.014	0.025
Temporal_Pole_Mid_R	7.891	0.005	1.74	0.022	0.017	0.014
Temporal_Inf_L	−11.084	0.001	−1.483	0.024	−0.013	0.019
Temporal_Inf_R			−1.407	0.034	−0.012	0.032

### Linear regression of FA value and age

**Figure 4:**
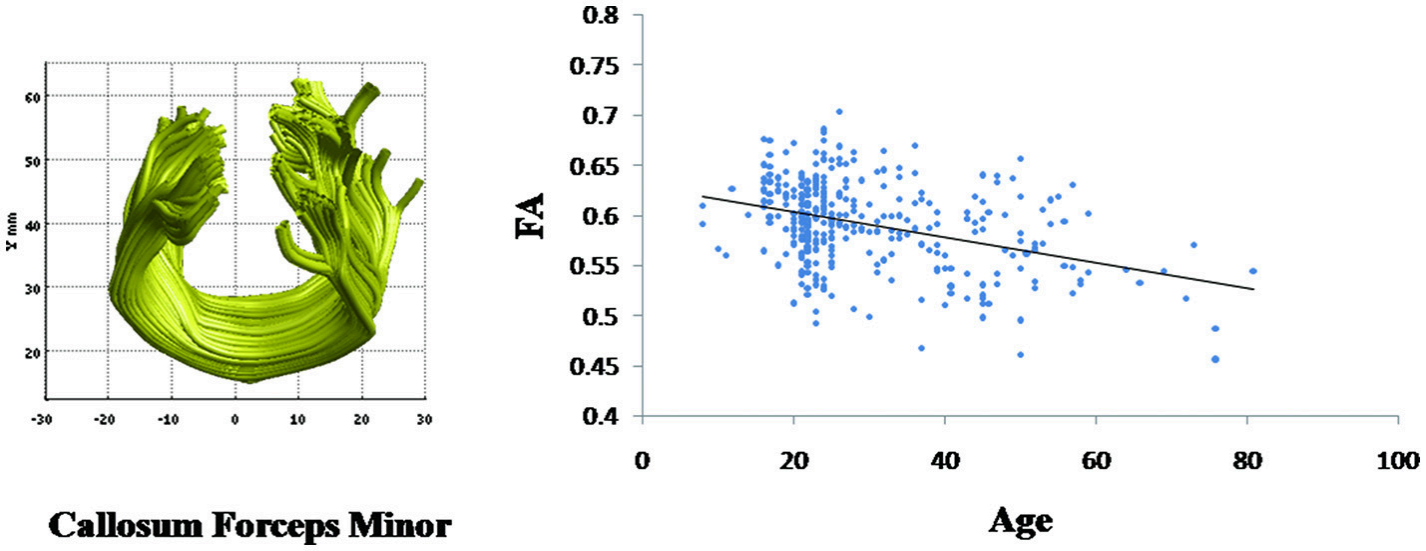
Change with age in the callosum forceps minor fasciculus fractional anisotropy (FA) values. Age is negatively correlated with the mean FA values for the callosum forceps minor (β = −0.001, *p* < 0.001).

Linear age-related decreases in the FA value were observed in callosum forceps minor (β = −0.001, *p* < 0.001) (Figure 4). However, the mean FA values of other fiber bundles did not demonstrate linear relationship with age.

### Linear regression of nodal topological properties and fasciculus structure connectivity

As observed in Supplementary Table 1, all of the mean FA values had either positive or negative association with node betweenness of 49 brain regions. Among these, only 14 showed direct positive anatomical relationship with fiber bundles, which were mainly located in the frontal and occipital lobes. Likewise, the mean FA values of 13 of the fiber bundles had either positive or negative linear relationship with node degree of 52 brain regions, while 14 of the fiber bundles had either positive or negative linear relationship with node efficiency in 60 brain regions. Among these regions, the node degree of 14 and the node efficiency of 16 had a direct anatomical relationship with the fiber bundles (Supplementary Table 2); these relationships were primarily negative, and located in the frontal and occipital lobes. We also observed that the long fiber bundles had significant relationships with more brain regions than the short fiber bundles.

## Discussion

In this study, we investigated age related changes in topological properties of brain functional network and structural connectivity. We observed that the nodes and topological properties that changed with increasing age were limited to specific regions, and FA values of most fiber bundles did not alter with increasing age, suggesting that age related changes are more pronounced in functional rather than structural connectivity measures indicating these measures do not have a direct one-to-one mapping. It was also noted that fiber bundles with longer fibers exhibited more pronounced effect on the properties of functional network rather than structural network.

For regional characteristics of functional properties, we observed that age related decreases in node betweenness were primarily located in the superior frontal lobe, right occipital lobe and global hubs. Among 21 brain regions that had significant relationship with age, 11 global hubs identified crucial to efficient communication (Achard et al., 2006; Chen et al., 2008; Iturria-Medina et al., 2008; Gong et al., 2009a; He et al., 2009; Wu et al., 2012) were mostly association cortices (13 out of 44) with no identifiable primary regions. This result supports the hypothesis that age-related changes are characteristics of association cortices as opposed to primary cortices (Albert, 1994). Decreases in local betweenness were primarily located in the superior frontal lobe and right occipital lobe, which is consistent with previous studies (Wu et al., 2012, 2013). Association regions contribute to the integrity of multiple functional systems, such, as memory and attention systems, and are mainly involved intelligent processing and maintenance of superior spiritual activity (Mesulam, 1998). Vulnerability of frontal regions with advancing age might explain the reason behind the cognitive function decline in many elderly populations (Jernigan et al., 2001). Moreover, identification of association cortex supports the hypothesis that age-related changes are the characteristics of association cortex but primary cortices (Park et al., 2008), which also in consistent with “last –in-first-out” hypothesis indicating that late-maturing regions (such as heteromodal association cortices) have damaging effects of aging (Kalpouzos et al., 2009; Terribilli et al., 2011). Age related increases in node degree and node efficiency were predominantly located in the posterior frontal lobe and parietal lobe. The alteration in nodal degree and nodal efficiency were very similar to each other. Nearly half of the 90 brain regions were observed to have changes with regard to these two nodal properties. Among these regions, alteration ratio of sub cortical (8 of 10), limbic (2 of 4), primary (4 of 8), and paralimbic (10 of 24) were similar, but alteration ratio of the association regions (16 of 44) in nodal degree was slightly larger than that of nodal efficiency (15 of 44), suggesting a tight relationship between these two regional nodal properties.

On the contrary to the functional studies, we found that the relationship between nodal topological properties and fasciculus structures were primarily positive for nodal betweenness, and negative for nodal degree and nodal efficiency. These brain regions also had both direct and indirect anatomical relationships with the 14 fiber bundles. Since, the normalized betweenness measures the ability of a node relative to information flow between other nodes within the network, a positive relationship between the fasciculus FA and node betweenness in most regions suggests that increased structural connectivity of the fasciculus may improve communication between a node and other nodes in the network. Likewise, both direct and indirect relationships with the 14 fiber bundles might be due to the co-activation of the regions even when there is no direct structural connection between them (Rubinov and Sporns, 2010), which is also shown by previous studies indicating strong relationship between structural integrity and functional connectivity in resting state networks direct one-to-one relationship between structural and functional connectivity (Bullmore and Sporns, 2009; Damoiseaux and Greicius, 2009).

Other finding of our study was that the FA values of most of the fiber bundles did not alter with increasing age except that of callosum forceps minor. This age-dependent decrease in FA in the callosum forceps minor was in accordance with findings from several recent studies on the age-related alterations in the white matter microstructure (Park et al., 2004; Salat et al., 2005; Ardekani et al., 2007). This alteration in the organization of the corpus callosum during the aging process may explain the reason behind aging to be vulnerable to neurodegenerative disorders such as in Alzheimer's disease, as there might be an interruption of information from the sensory neocortex to the prefrontal neocortex. However, a study by Voineskos et al. (2012), healthy individuals (*n* = 48) age ranging from 18 to 85 indicated an anterioposterior gradient of age related decline in corpus callosum fibers, where a potential role of regional white matter damage (i.e., posterior fibers of the corpus callosum) in influencing different cognitive performances in healthy subjects was noted. One of the reasons for different findings might be due to difference in sample size, where a larger sample size might have permitted retention of more paths in their study. In addition, larger number of sample subjects between the age group 20 and 40 in our study might also play its role in different findings between these two studies. Other reason includes the methodological differences in these studies where Voineskos and colleagues did not study all white matter tracts (focusing primarily on cortico-cortical white matter tracts). The current study also indicates that the FA value of the most of the fiber bundles did not substantially change with age. However, a direct inference on anatomical connectivity differences cannot be based on this observation alone. The unchanged FA value might also be due to a balance created among alterations in fiber size, density, and myelination or fiber coherence. However, these differences in findings also suggest that white matter alterations are variable throughout the brain.

Apart from the findings above, functional brain network exhibited economical small-world properties in all healthy individuals. In this study, we demonstrated that there was increase in global efficiency (β = 0.009, *p* = 0.004), local efficiency (β = 0.01, *p* = 0.002), and clustering coefficient (β = 0.011, *p* = 0.002) (Figure 2) in functional brain network with the advancing age. An economical small-world offers a topological substrate for specialized or modular processing in local neighborhoods and distributed or integrated processing over the entire network with the combination of both high clustering and low characteristic path length (Sporns and Zwi, 2004; Stam, 2004; Achard et al., 2006; Achard and Bullmore, 2007). Small-world properties have been demonstrated by the studies using fMRI in the human brain functional networks (Eguiluz et al., 2005; Salvador et al., 2005; Achard et al., 2006; He et al., 2007)and have also demonstrated that these properties are the characteristics of large-scale anatomical networks of the human cerebral cortex. Thus, our findings also support the notion that there is the presence of an efficient network structure across the development process.

## Limitations

Several issues must be addressed while considering the results of our study. First, the population distribution of our study was not uniform. Proportions of subjects between 20 and 40 year was larger than that of other age groups. Second, we used a DTI-based streamline tractography approach (Mori et al., 1999; Basser et al., 2000) to define the edges of the structural network. Although, this is the most widely applied tractography method primarily due to its simplicity, robustness, and speed (Cheng et al., 2012; Griffa et al., 2013), such tractography method, should be used cautiously in order to resolve crossing fiber bundles (Tournier et al., 2011; Jones et al., 2013). Third, due to limited number of subjects with neuropsychological assessment in our study, we were restricted in determining the relationship between white matter tracts and cognitive ratings in our findings. Further studies will benefit from the use of assessment scales to quantify the association between white matter integrity and cognitive performance. Finally, we adopted Automated Anatomical Labeling (AAL) template as a parcellation scheme. The AAL template is based on sulcal patterns from only one subject. Primary advantage of using AAL template for nodal parcellation is that, it can support a direct comparison of results to previous connectome studies using the same AAL template in healthy adults (Gong et al., 2009a) and patient populations (Zalesky et al., 2011). It is important to consider that although the atlas that we used was carefully checked for registration errors, a probabilistic atlas of the human brain might be better for obtaining regional parcellation or defining individual brain regions through a combination of DTI with fMRI due to the inter-individual variability of anatomical structures (Sporns et al., 2005).

## Conclusion

Current study indicates that, age related changes are more pronounced in the functional than in structural measure indicating these measures do not have a direct one-to-one mapping. Frontal regions are more vulnerable with advancing age and fiber bundles with longer fibers exhibited pronounced effect on the functional network properties. Future longitudinal studies would be useful to investigate the changes in the functional and anatomical neural networks that occur with normal aging.

## Author contributions

Study concept and design: SL and QG. Acquisition, analysis, or interpretation of data: HS and JiaL. Drafting of the manuscript: CS and JiaL. Critical revision of the manuscript for important intellectual content: SL and PL. Statistical analysis: HS, JieL, and WZ. Obtained funding: SL and QG. Adminstrative, technical, or material support: CS, YX, YZ, and LY. Study supervision: SL.

### Conflict of interest statement

The authors declare that the research was conducted in the absence of any commercial or financial relationships that could be construed as a potential conflict of interest.
