# Supplementary material for: Age Related Changes in Topological Properties of Brain Functional Network and Structural Connectivity
Source: Front Neurosci. 2018 May 15;12:318. doi: 10.3389/fnins.2018.00318 (PMC5962656; doi:10.3389/fnins.2018.00318)
Supplement: Supplementary file 1 [file Table_1.DOCX]

Supplementary Table -1: Regression models assessing the effect of FA on nodal topological properties

| Fiber bundles | Brain Regions | Betweenness | |  | Degree | |  | Nodeefficiency | |
| --- | --- | --- | --- | --- | --- | --- | --- | --- | --- |
|  |  | Beta | p-Value |  | Beta | p-Value |  | Beta | p-Value |
| L Thalamic Radiation | Frontal_Mid_L |  |  |  | -553.861 | 0.046 |  |  |  |
|  | Frontal_Mid_R |  |  |  | -662.673 | 0.025 |  | -4.881 | 0.046 |
|  | Rolandic_Oper_L | 3892.845 | 0.009 |  | 617.920 | 0.019 |  | 4.882 | 0.023 |
|  | Supp_Motor_Area_L | -2585.718 | 0.017 |  |  |  |  |  |  |
|  | Olfactory_L | 2400.436 | 0.015 |  |  |  |  |  |  |
|  | Rectus_L |  |  |  | -587.751 | 0.043 |  |  |  |
|  | Cingulum_Mid_R |  |  |  | -840.158 | 0.005 |  | -6.299 | 0.010 |
|  | Calcarine_R | 3661.658 | 0.001 |  |  |  |  |  |  |
|  | Lingual_L |  |  |  | -909.601 | 0.006 |  | -2.272 | 0.005 |
|  | Lingual_R |  |  |  | -736.257 | 0.023 |  | -5.996 | 0.024 |
|  | Occipital_Sup_R |  |  |  | -774.883 | 0.029 |  | -6.204 | 0.035 |
|  | Occipital_Inf_L |  |  |  | -768.658 | 0.020 |  | -5.871 | 0.034 |
|  | Occipital_Inf_R |  |  |  | -885.710 | 0.008 |  | -6.417 | 0.023 |
|  | Fusiform_L |  |  |  | -701.339 | 0.021 |  | -5.932 | 0.020 |
|  | Fusiform_R |  |  |  | -784.356 | 0.013 |  | -6.150 | 0.020 |
|  | Postcentral_R |  |  |  | -841.858 | 0.011 |  | -6.796 | 0.013 |
|  | SupraMarginal_L | 3627.297 | 0.008 |  | 629.984 | 0.034 |  | 5.728 | 0.021 |
|  | Angular_R |  |  |  | -629.592 | 0.027 |  | -4.938 | 0.035 |
|  | Paracentral_Lobule_R |  |  |  | -718.355 | 0.017 |  |  |  |
|  | Putamen_R | 4152.638 | 0.004 |  |  |  |  |  |  |
|  | Pallidum_L |  |  |  | -707.915 | 0.016 |  | -4.967 | 0.039 |
|  | Pallidum_R |  |  |  | -596.532 | 0.048 |  |  |  |
|  | Thalamus_L |  |  |  | -1145.660 | <0.001 |  | -9.664 | <0.001 |
|  | Thalamus_R | -2213.997 | 0.043 |  | -1133.140 | 0.001 |  | -9.369 | 0.001 |
|  | Temporal_Sup_L | 2975.550 | 0.011 |  | 577.549 | 0.025 |  |  |  |
| R Thalamic Radiation | Precentral_R | -3370.681 | 0.026 |  | -796.906 | 0.024 |  | -5.969 | 0.038 |
|  | Frontal_Mid_L | -617.566 | 0.050 |  |  |  |  |  |  |
|  | Frontal_Sup_Medial_R |  |  |  | -653.257 | 0.040 |  |  |  |
|  | Cingulum_Mid_R |  |  |  | -791.417 | 0.021 |  | -6.734 | 0.016 |
|  | Hippocampus_L | -2467.474 | 0.049 |  | -621.025 | 0.030 |  |  |  |
|  | Amygdala_L |  |  |  | -676.500 | 0.024 |  | -5.476 | 0.025 |
|  | Calcarine_R | 3193.014 | 0.011 |  |  |  |  |  |  |
|  | Lingual_L |  |  |  | -1035.61 | 0.006 |  | -9.040 | 0.003 |
|  | Lingual_R |  |  |  | -790.284 | 0.033 |  | -6.845 | 0.023 |
|  | Occipital_Sup_L |  |  |  | -808.019 | 0.037 |  | -6.900 | 0.032 |
|  | Occipital_Sup_R |  |  |  | -973.285 | 0.016 |  | -8.501 | 0.011 |
|  | Occipital_Mid_R |  |  |  | -793.857 | 0.026 |  | -5.907 | 0.046 |
|  | Occipital_Inf_L |  |  |  | -990.137 | 0.008 |  | -8.540 | 0.007 |
|  | Occipital_Inf_R |  |  |  | -1275.690 | 0.001 |  | -10.158 | 0.001 |
|  | Fusiform_L |  |  |  | -1011.180 | 0.003 |  | -8.873 | 0.002 |
|  | Fusiform_R |  |  |  | -1059.720 | 0.003 |  | -9.163 | 0.002 |
|  | Postcentral_R |  |  |  | -1065.870 | 0.004 |  | -8.890 | 0.004 |
|  | SupraMarginal_L | 5628.677 | <0.001 |  |  |  |  |  |  |
|  | Angular_R |  |  |  | -837.847 | 0.009 |  | -6.953 | 0.009 |
|  | Paracentral_Lobule_R |  |  |  | -760.113 | 0.026 |  | -5.674 | 0.048 |
|  | Putamen_R | 4746.995 | 0.004 |  |  |  |  |  |  |
|  | Pallidum_L |  |  |  | -942.636 | 0.005 |  | -7.351 | 0.007 |
|  | Thalamus_L |  |  |  | -1067.850 | 0.003 |  | -9.914 | 0.001 |
|  | Thalamus_R |  |  |  | -1220.300 | 0.001 |  | -10.568 | 0.001 |
|  | Temporal_Sup_L | 3162.304 | 0.018 |  |  |  |  |  |  |
|  | Temporal_Mid_L |  |  |  | -631.308 | 0.026 |  | -5.330 | 0.027 |
| L Corticospinal | Frontal_Sup_Orb_R | 4089.760 | 0.001 |  |  |  |  |  |  |
|  | Frontal_Inf_Orb_L | 7285.718 | 0.001 |  |  |  |  |  |  |
|  | Rectus_R | -3817.654 | 0.020 |  |  |  |  |  |  |
|  | Amygdala_L |  |  |  | -785.887 | 0.042 |  |  |  |
|  | Lingual_L | -3962.734 | 0.015 |  |  |  |  |  |  |
|  | Postcentral_L | 3348.897 | 0.048 |  |  |  |  |  |  |
|  | SupraMarginal_L | 6551.595 | 0.001 |  |  |  |  |  |  |
|  | Angular_R | -6360.235 | 0.009 |  |  |  |  |  |  |
|  | Pallidum_L | -3335.054 | 0.029 |  | -991.312 | 0.022 |  | -7.855 | 0.026 |
| R Corticospinal | Cingulum_Mid_L | -3107.915 | 0.028 |  |  |  |  |  |  |
|  | ParaHippocampal_L |  |  |  |  |  |  | 5.663 | 0.050 |
|  | ParaHippocampal_R |  |  |  |  |  |  | 5.936 | 0.036 |
|  | SupraMarginal_L | 4819.004 | 0.011 |  |  |  |  |  |  |
|  | Pallidum_L | -3540.277 | 0.014 |  |  |  |  |  |  |
|  | Heschl_L |  |  |  |  |  |  | 6.399 | 0.037 |
| Callosum Forceps Major | Frontal_Sup_L |  |  |  |  |  |  | 3.750 | 0.033 |
|  | Frontal_Mid_Orb_L | -1917.708 | 0.034 |  |  |  |  |  |  |
|  | Frontal_Sup_Medial_R | 2756.272 | 0.027 |  |  |  |  |  |  |
|  | Pallidum_L |  |  |  | -492.747 | 0.042 |  |  |  |
|  | Heschl_R | -1828.404 | 0.026 |  |  |  |  |  |  |
|  | Temporal_Pole_Sup_R | 2431.194 | 0.031 |  |  |  |  |  |  |
| Callosum Forceps Minor | Frontal_Sup_L | 2451.512 | 0.036 |  |  |  |  |  |  |
|  | Frontal_Mid_L |  |  |  | -573.789 | 0.024 |  | -4.356 | 0.036 |
|  | Frontal_Mid_R |  |  |  | -545.889 | 0.044 |  |  |  |
|  | Rolandic_Oper_L | 3647.825 | 0.007 |  |  |  |  |  |  |
|  | Insula_L | 4351.967 | 0.013 |  |  |  |  |  |  |
|  | Cingulum_Mid_L | -1879.130 | 0.044 |  | -986.564 | <0.001 |  | -7.566 | 0.001 |
|  | Cingulum_Mid_R |  |  |  | -1130.05 | <0.001 |  | -8.911 | <0.001 |
|  | ParaHippocampal_L |  |  |  | -572.741 | 0.012 |  | -4.763 | 0.013 |
|  | Amygdala_L |  |  |  | -607.644 | 0.012 |  | -4.895 | 0.013 |
|  | Calcarine_R | 3070.377 | 0.002 |  |  |  |  |  |  |
|  | Cuneus_R |  |  |  | -657.113 | 0.019 |  | -5.336 | 0.018 |
|  | Lingual_L |  |  |  | -786.295 | 0.009 |  | -6.723 | 0.007 |
|  | Lingual_R |  |  |  | -703.343 | 0.018 |  | -5.729 | 0.018 |
|  | Occipital_Sup_L |  |  |  | -1014.990 | 0.001 |  | -7.918 | 0.002 |
|  | Occipital_Sup_R |  |  |  | -1000.650 | 0.002 |  | -7.962 | 0.003 |
|  | Occipital_Mid_R |  |  |  | -625.910 | 0.029 |  |  |  |
|  | Occipital_Inf_L |  |  |  | -1044.76 | 0.001 |  | -8.289 | 0.001 |
|  | Occipital_Inf_R |  |  |  | -1062.350 | <0.001 |  | -8.055 | 0.002 |
|  | Fusiform_L |  |  |  | -1096.530 | <0.001 |  | -8.926 | <0.001 |
|  | Fusiform_R |  |  |  | -869.828 | 0.002 |  | -7.194 | 0.003 |
|  | Postcentral_R |  |  |  | -637.641 | 0.035 |  | -5.229 | 0.037 |
|  | Parietal_Sup_L |  |  |  | -700.995 | 0.006 |  | -5.098 | 0.014 |
|  | Caudate_L |  |  |  | -613.853 | 0.014 |  | -4.252 | 0.040 |
|  | Putamen_L | 3281.442 | 0.013 |  | -552.565 | 0.027 |  | -4.533 | 0.024 |
|  | Pallidum_L |  |  |  | -916.284 | 0.001 |  | -7.314 | 0.001 |
|  | Thalamus_L |  |  |  | -946.606 | 0.001 |  | -8.299 | 0.001 |
|  | Thalamus_R |  |  |  | -986.983 | 0.001 |  | -8.123 | 0.002 |
|  | Temporal_Sup_R | -2638.377 | 0.016 |  |  |  |  |  |  |
| L IFOF | Frontal_Sup_Orb_L | 2387.511 | 0.044 |  |  |  |  |  |  |
|  | Frontal_Sup_Orb_R | 3052.800 | 0.003 |  |  |  |  |  |  |
|  | Rolandic_Oper_L | 3295.521 | 0.020 |  |  |  |  |  |  |
|  | Rectus_L | -3281.069 | 0.010 |  | -790.085 | 0.004 |  | -6.294 | 0.004 |
|  | Calcarine_R | 3712.140 | <0.001 |  |  |  |  |  |  |
|  | Caudate_R | 1843.406 | 0.044 |  |  |  |  |  |  |
|  | Putamen_L | 3049.950 | 0.012 |  |  |  |  |  |  |
|  | Putamen_R | 2955.677 | 0.033 |  |  |  |  |  |  |
|  | Thalamus_L |  |  |  |  |  |  | -5.494 | 0.036 |
|  | Temporal_Pole_Sup_R |  |  |  | -552.098 | 0.043 |  |  |  |
|  | Temporal_Mid_L |  |  |  | -714.666 | 0.003 |  | -5.916 | 0.004 |
| R IFOF | Frontal_Sup_Orb_L | 2858.097 | 0.028 |  | 543.081 | 0.049 |  |  |  |
|  | Frontal_Sup_Orb_R | 2335.837 | 0.041 |  | 569.684 | 0.049 |  |  |  |
|  | Frontal_Inf_Oper_L |  |  |  |  |  |  | -4.262 | 0.005 |
|  | Frontal_Inf_Oper_R |  |  |  |  |  |  | -3.697 | 0.034 |
|  | Frontal_Inf_Tri_L |  |  |  |  |  |  | -4.640 | 0.002 |
|  | Frontal_Inf_Tri_R |  |  |  |  |  |  | -3.684 | 0.025 |
|  | Frontal_Inf_Orb_L |  |  |  |  |  |  | -3.687 | 0.002 |
|  | Frontal_Inf_Orb_R |  |  |  |  |  |  | -4.166 | 0.019 |
|  | Rolandic_Oper_L | 3161.162 | 0.044 |  |  |  |  |  |  |
|  | Supp_Motor_Area_L | 2772.764 | 0.013 |  |  |  |  | -3.501 | 0.043 |
|  | Rectus_L | -3386.522 | 0.015 |  |  |  |  |  |  |
|  | Cingulum_Mid_L |  |  |  |  |  |  | -3.758 | 0.037 |
|  | Cingulum_Post_R |  |  |  |  |  |  | 3.868 | 0.032 |
|  | Amygdala_R |  |  |  |  |  |  | -3.250 | 0.044 |
|  | Calcarine_L |  |  |  |  |  |  | -4.209 | 0.020 |
|  | Calcarine_R | 2991.610 | 0.010 |  |  |  |  |  |  |
|  | Lingual_L |  |  |  |  |  |  | -4.979 | 0.014 |
|  | Lingual_R |  |  |  |  |  |  | -4.386 | 0.027 |
|  | Occipital_Sup_R |  |  |  |  |  |  | -5.068 | 0.021 |
|  | Occipital_Inf_L |  |  |  |  |  |  | -7.524 | <0.001 |
|  | Occipital_Inf_R |  |  |  |  |  |  | -5.648 | 0.007 |
|  | Fusiform_L |  |  |  | -690.390 | 0.031 |  |  |  |
|  | Parietal_Sup_L | -4422.169 | <0.001 |  | -590.762 | 0.043 |  |  |  |
|  | SupraMarginal_L | 3531.672 | 0.015 |  |  |  |  |  |  |
|  | Caudate_L |  |  |  |  |  |  | -5.021 | 0.003 |
|  | Putamen_L |  |  |  |  |  |  | -3.277 | 0.046 |
|  | Pallidum_L |  |  |  |  |  |  | -3.978 | 0.027 |
|  | Temporal_Sup_L | 2926.343 | 0.018 |  | 609.722 | 0.020 |  |  |  |
|  | Temporal_Pole_Sup_R |  |  |  |  |  |  | -3.859 | 0.031 |
|  | Temporal_Mid_L |  |  |  | -654.257 | 0.010 |  | -5.517 | <0.001 |
|  | Temporal_Pole_Mid_R |  |  |  |  |  |  | -4.295 | 0.030 |
|  | Temporal_Inf_L |  |  |  |  |  |  | -6.748 | <0.001 |
|  | Temporal_Inf_R |  |  |  |  |  |  | -4.870 | 0.004 |
| L ILF | Frontal_Sup_R | 2646.594 | 0.030 |  | 736.692 | 0.006 |  | 5.824 | 0.008 |
|  | Frontal_Sup_Orb_L | 2700.966 | 0.017 |  | 527.434 | 0.033 |  | 4.698 | 0.022 |
|  | Frontal_Sup_Orb_R | 2602.766 | 0.009 |  | 707.201 | 0.006 |  | 6.581 | 0.002 |
|  | Rolandic_Oper_L | 4206.630 | 0.002 |  |  |  |  |  |  |
|  | Olfactory_L |  |  |  | 548.999 | 0.025 |  | 5.052 | 0.016 |
|  | Olfactory_R | 2796.416 | 0.002 |  | 780.120 | 0.001 |  | 6.553 | 0.002 |
|  | Frontal_Sup_Medial_L | -2978.253 | 0.020 |  |  |  |  |  |  |
|  | ParaHippocampal_R | -2541.571 | 0.042 |  |  |  |  |  |  |
|  | Calcarine_R | 2515.162 | 0.013 |  |  |  |  |  |  |
|  | Lingual_R | 2534.918 | 0.021 |  |  |  |  |  |  |
|  | Occipital_Mid_R |  |  |  | -816.394 | 0.005 |  | -6.491 | 0.007 |
|  | Fusiform_L |  |  |  | -580.393 | 0.038 |  | -4.733 | 0.044 |
|  | Parietal_Sup_L | -2734.767 | 0.011 |  |  |  |  |  |  |
|  | Precuneus_L |  |  |  | 505.848 | 0.032 |  | 4.041 | 0.037 |
|  | Caudate_R | 2059.013 | 0.017 |  |  |  |  |  |  |
|  | Pallidum_L |  |  |  | -627.298 | 0.021 |  | -4.781 | 0.031 |
|  | Thalamus_L | -2255.369 | 0.026 |  | -821.001 | 0.005 |  | -6.988 | 0.006 |
|  | Thalamus_R | -2516.641 | 0.012 |  | -678.033 | 0.025 |  | -5.698 | 0.028 |
| R ILF | Frontal_Sup_R |  |  |  | 683.935 | 0.016 |  | 5.327 | 0.021 |
|  | Frontal_Sup_Orb_L | 2568.744 | 0.032 |  | 965.201 | <0.001 |  | 8.168 | <0.001 |
|  | Frontal_Sup_Orb_R |  |  |  | 812.054 | 0.003 |  | 7.039 | 0.002 |
|  | Olfactory_L |  |  |  | 581.293 | 0.024 |  | 4.627 | 0.037 |
|  | Olfactory_R | 2309.438 | 0.016 |  | 728.112 | 0.005 |  | 5.768 | 0.009 |
|  | Rectus_L | -2729.789 | 0.034 |  |  |  |  |  |  |
|  | Amygdala_R | 3078.827 | 0.009 |  | 678.937 | 0.007 |  | 5.941 | 0.004 |
|  | Calcarine_R | 3909.348 | <0.001 |  |  |  |  |  |  |
|  | Lingual_R | 2482.920 | 0.032 |  |  |  |  |  |  |
|  | Occipital_Sup_R | -2965.608 | 0.022 |  |  |  |  |  |  |
|  | Occipital_Inf_L |  |  |  | -575.580 | 0.018 |  | -6.520 | 0.016 |
|  | Occipital_Inf_R |  |  |  | -713.366 | 0.028 |  | -6.334 | 0.021 |
|  | Fusiform_L |  |  |  | -601.768 | 0.042 |  |  |  |
|  | Parietal_Sup_L | -2744.739 | 0.016 |  |  |  |  |  |  |
|  | Putamen_R | 3475.208 | 0.013 |  |  |  |  |  |  |
|  | Thalamus_R | -2307.790 | 0.030 |  |  |  |  |  |  |
|  | Temporal_Sup_L | 2346.447 | 0.040 |  |  |  |  |  |  |
|  | Temporal_Mid_L |  |  |  | -500.375 | 0.039 |  | -4.115 | 0.047 |
| L SLF | Frontal_Sup_R |  |  |  | 620.426 | 0.019 |  | 5.645 | 0.010 |
|  | Olfactory_R | 1912.361 | 0.040 |  | 540.445 | 0.031 |  | 4.224 | 0.048 |
|  | Cingulum_Mid_L | -3443.997 | <0.001 |  | -836.803 | 0.003 |  | -6.274 | 0.006 |
|  | Cingulum_Mid_R | -2416.917 | 0.029 |  | -822.528 | 0.004 |  | -6.306 | 0.006 |
|  | Calcarine_L | 2318.773 | 0.019 |  |  |  |  |  |  |
|  | Occipital_Mid_R | -2543.731 | 0.014 |  | -601.264 | 0.041 |  |  |  |
|  | Postcentral_L | 2238.002 | 0.039 |  |  |  |  |  |  |
|  | Caudate_R | 2062.070 | 0.021 |  |  |  |  |  |  |
|  | Heschl_R |  |  |  | -494.578 | 0.048 |  |  |  |
| R SLF | Frontal_Mid_L | 3702.172 | 0.016 |  |  |  |  | 4.453 | 0.042 |
|  | Frontal_Inf_Oper_R | -2889.579 | 0.034 |  |  |  |  |  |  |
|  | Frontal_Inf_Tri_L | -2092.494 | 0.037 |  |  |  |  |  |  |
|  | Hippocampus_L | -2136.430 | 0.045 |  |  |  |  |  |  |
|  | Occipital_Sup_R |  |  |  |  |  |  | 6.319 | 0.027 |
|  | Occipital_Inf_R | 2029.505 | 0.033 |  | 870.535 | 0.007 |  | 8.314 | 0.002 |
|  | Paracentral_Lobule_L | 2169.029 | 0.038 |  |  |  |  |  |  |
|  | Putamen_R | -4152.113 | 0.003 |  |  |  |  |  |  |
|  | Heschl_L | 2036.735 | 0.035 |  | 506.505 | 0.040 |  | 4.507 | 0.033 |
| L Uncinate | Precentral_L | 1862.617 | 0.027 |  | 461.287 | 0.024 |  | 3.868 | 0.019 |
|  | Frontal_Sup_R |  |  |  | 457.367 | 0.028 |  | 3.813 | 0.024 |
|  | Frontal_Sup_Orb_L |  |  |  | 422.365 | 0.024 |  | 3.562 | 0.022 |
|  | Frontal_Sup_Orb_R | 2433.036 | 0.001 |  | 437.486 | 0.027 |  | 4.194 | 0.01 |
|  | Rolandic_Oper_L | 2260.582 | 0.032 |  |  |  |  |  |  |
|  | Supp_Motor_Area_R |  |  |  | -414.987 | 0.043 |  |  |  |
|  | Olfactory_L |  |  |  | 392.586 | 0.037 |  |  |  |
|  | Olfactory_R | 1450.402 | 0.038 |  | 458.179 | 0.015 |  | 3.674 | 0.022 |
|  | Rectus_L | -2022.204 | 0.029 |  | -515.130 | 0.011 |  | -3.837 | 0.018 |
|  | Insula_R |  |  |  | -376.990 | 0.041 |  |  |  |
|  | Cingulum_Mid_L | -1754.167 | 0.015 |  |  |  |  |  |  |
|  | ParaHippocampal_R | -1899.410 | 0.048 |  | -378.966 | 0.026 |  | -3.153 | 0.028 |
|  | Cuneus_L |  |  |  | -488.615 | 0.023 |  | -3.823 | 0.027 |
|  | Cuneus_R |  |  |  | -598.553 | 0.006 |  | -5.092 | 0.003 |
|  | Occipital_Mid_R |  |  |  | -623.994 | 0.005 |  | -4.849 | 0.009 |
|  | Fusiform_L |  |  |  | -485.353 | 0.024 |  | -4.092 | 0.023 |
|  | Postcentral_L | 1653.148 | 0.043 |  |  |  |  |  |  |
|  | SupraMarginal_L | 2322.786 | 0.017 |  |  |  |  |  |  |
|  | Paracentral_Lobule_L |  |  |  |  |  |  | 3.755 | 0.049 |
|  | Caudate_R | 1580.178 | 0.019 |  |  |  |  |  |  |
|  | Paracentral_Lobule_L |  |  |  |  |  |  | 3.755 | 0.049 |
|  | Putamen_L | 2189.976 | 0.014 |  |  |  |  |  |  |
|  | Pallidum_L |  |  |  | -470.194 | 0.024 |  | -3.468 | 0.042 |
|  | Pallidum_R |  |  |  | -434.823 | 0.042 |  |  |  |
|  | Temporal_Mid_L |  |  |  | -441.342 | 0.012 |  | -4.109 | 0.006 |
| R Uncinate | Frontal_Sup_Orb_R | 2103.857 | 0.045 |  |  |  |  |  |  |
|  | Frontal_Mid_Orb_L |  |  |  | -561.884 | 0.028 |  | -4.111 | 0.047 |
|  | Rolandic_Oper_L | 4117.323 | 0.004 |  |  |  |  |  |  |
|  | Supp_Motor_Area_L |  |  |  | -595.201 | 0.031 |  | -4.481 | 0.046 |
|  | Supp_Motor_Area_R |  |  |  | -749.923 | 0.007 |  | -5.529 | 0.017 |
|  | Olfactory_R | 2608.757 | 0.006 |  |  |  |  |  |  |
|  | Rectus_L | -2552.629 | 0.048 |  | -974.047 | 0.001 |  | -7.178 | 0.001 |
|  | Rectus_R |  |  |  | -633.203 | 0.021 |  | -4.434 | 0.037 |
|  | Insula_R |  |  |  | -669.736 | 0.008 |  | -5.062 | 0.014 |
|  | Cingulum_Ant_R | 2948.495 | 0.037 |  |  |  |  |  |  |
|  | Cingulum_Mid_L | -2629.151 | 0.008 |  | -907.982 | 0.002 |  | -7.194 | 0.002 |
|  | Cingulum_Mid_R |  |  |  | -847.382 | 0.004 |  | -6.588 | 0.006 |
|  | ParaHippocampal_R | -2664.658 | 0.044 |  |  |  |  |  |  |
|  | Amygdala_R | 2735.629 | 0.021 |  |  |  |  |  |  |
|  | Calcarine_R | 3407.391 | 0.001 |  |  |  |  |  |  |
|  | Occipital_Mid_R |  |  |  | -691.825 | 0.023 |  | -5.333 | 0.035 |
|  | Fusiform_L |  |  |  | -622.207 | 0.035 |  | -5.476 | 0.027 |
|  | Parietal_Sup_L |  |  |  | -591.952 | 0.028 |  |  |  |
|  | Paracentral_Lobule_L | 2115.115 | 0.044 |  |  |  |  |  |  |
|  | Putamen_L | 2711.018 | 0.027 |  |  |  |  |  |  |
|  | Temporal_Pole_Sup_R |  |  |  | -610.563 | 0.027 |  | -4.999 | 0.033 |

Ant=anterior; IFOF= inferior fronto-occipital fasciculus; ILF=inferior longitudinal fasciculus; Inf=Inferior; L= left; Mid=middle; Orb=orbital; Oper=opercular; Post=posterior; R=right; Sup=superior; SLF= superior longitudinal fasciculus.
