# Supplementary material for: Age Related Changes in Topological Properties of Brain Functional Network and Structural Connectivity
Source: Front Neurosci. 2018 May 15;12:318. doi: 10.3389/fnins.2018.00318 (PMC5962656; doi:10.3389/fnins.2018.00318)
Supplement: Supplementary file 2 [file Table_2.DOCX]

Supplementary Table 2: Brain regions had a direct anatomic relationship with the fiber bundles

| Fiber bundles | Brain Regions | Betweenness | |  | Degree | |  | Nodeefficiency | |
| --- | --- | --- | --- | --- | --- | --- | --- | --- | --- |
|  |  | Beta | p-Value |  | Beta | p-Value |  | Beta | p-Value |
| L Thalamic Radiation | Frontal_Mid_L |  |  |  | -553.861 | 0.046 |  |  |  |
|  | Rolandic_Oper_L | 3892.845 | 0.009 |  | 617.920 | 0.019 |  | 4.882 | 0.023 |
|  | Supp_Motor_Area_L | -2585.718 | 0.017 |  |  |  |  |  |  |
|  | Olfactory_L | 2400.436 | 0.015 |  |  |  |  |  |  |
|  | Rectus_L |  |  |  | -587.751 | 0.043 |  |  |  |
|  | Thalamus_L |  |  |  | -1145.660 | <0.001 |  | -9.664 | <0.001 |
| R Thalamic Radiation | Precentral_R | -3370.681 | 0.026 |  | -796.906 | 0.024 |  | -5.969 | 0.038 |
|  | Frontal_Sup_Medial_R |  |  |  | -653.257 | 0.040 |  |  |  |
|  | Postcentral_R |  |  |  | -1065.870 | 0.004 |  | -8.890 | 0.004 |
|  | Thalamus_R |  |  |  | -1220.300 | 0.001 |  | -10.568 | 0.001 |
| L Corticospinal | Postcentral_L | 3348.897 | 0.048 |  |  |  |  |  |  |
| Callosum Forceps Minor | Frontal_Sup_L | 2451.512 | 0.036 |  |  |  |  |  |  |
|  | Frontal_Mid_L |  |  |  | -573.789 | 0.024 |  | -4.356 | 0.036 |
|  | Frontal_Mid_R |  |  |  | -545.889 | 0.044 |  |  |  |
| L IFOF | Rectus_L | -3281.069 | 0.010 |  | -790.085 | 0.004 |  | -6.294 | 0.004 |
| R IFOF | Frontal_Inf_Oper_R |  |  |  |  |  |  | -3.697 | 0.034 |
|  | Frontal_Inf_Tri_R |  |  |  |  |  |  | -3.684 | 0.025 |
|  | Frontal_Inf_Orb_R |  |  |  |  |  |  | -4.166 | 0.019 |
|  | Calcarine_R | 2991.610 | 0.010 |  |  |  |  |  |  |
|  | Lingual_R |  |  |  |  |  |  | -4.386 | 0.027 |
|  | Occipital_Sup_R |  |  |  |  |  |  | -5.068 | 0.021 |
|  | Occipital_Inf_R |  |  |  |  |  |  | -5.648 | 0.007 |
| L ILF | Fusiform_L |  |  |  | -580.393 | 0.038 |  | -4.733 | 0.044 |
| R ILF | Lingual_R | 2482.920 | 0.032 |  |  |  |  |  |  |
|  | Occipital_Sup_R | -2965.608 | 0.022 |  |  |  |  |  |  |
|  | Occipital_Inf_R |  |  |  | -713.366 | 0.028 |  | -6.334 | 0.021 |
| R SLF | Frontal_Inf_Oper_R | -2889.579 | 0.034 |  |  |  |  |  |  |
|  | Occipital_Sup_R |  |  |  |  |  |  | 6.319 | 0.027 |
|  | Occipital_Inf_R | 2029.505 | 0.033 |  | 870.535 | 0.007 |  | 8.314 | 0.002 |
| L Uncinate | Olfactory_L |  |  |  | 392.586 | 0.037 |  |  |  |
|  | Rectus_L | -2022.204 | 0.029 |  | -515.130 | 0.011 |  | -3.837 | 0.018 |
| R Uncinate | Olfactory_R | 2608.757 | 0.006 |  |  |  |  |  |  |
|  | Rectus_R |  |  |  | -633.203 | 0.021 |  | -4.434 | 0.037 |
|  | Amygdala_R | 2735.629 | 0.021 |  |  |  |  |  |  |
|  | Temporal_Pole_Sup_R |  |  |  | -610.563 | 0.027 |  | -4.999 | 0.033 |

IFOF= inferior fronto-occipital fasciculus; ILF=inferior longitudinal fasciculus; Inf=Inferior; L= left; Mid=middle; Orb=orbital; Oper=opercular; Post=posterior; R=right; Sup=superior; SLF= superior longitudinal fasciculus.
